# Severe Portal Vein Thrombosis During Eltrombopag Treatment Concomitant Splenectomy for Immune Thrombocytopenia

**DOI:** 10.7759/cureus.17478

**Published:** 2021-08-27

**Authors:** Makoto Saito, Masanobu Morioka, Koh Izumiyama, Akio Mori, Takeshi Kondo

**Affiliations:** 1 Internal Medicine and Hematology, Aiiku Hospital, Sapporo, JPN

**Keywords:** immune thrombocytopenia, eltrombopag, splenectomy, portal vein thrombosis, case report

## Abstract

The treatment of immune thrombocytopenia (ITP) has recently changed; however, each treatment has not only advantages, but also disadvantages, and may have unexpected complications. We describe an instructive case of ITP that was complicated by severe portal vein thrombosis during treatment with eltrombopag, an oral thrombopoietin-receptor agonist (TPO-RA) drug, plus prednisolone (PSL) concomitant splenectomy. A male ITP patient who had been receiving eltrombopag treatment for more than four years at our department underwent a splenectomy at the age of 51. Soon after splenectomy, splenic vein and portal vein thrombosis developed, while splenectomy was ineffective. The patient resumed eltrombopag treatment after thrombosis disappeared. Although fluctuations in PLT were observed, eltrombopag and PSL were used together for a while. Subsequently, lower-limb deep vein thrombosis recurred, and edoxaban tosylate was administered for a total of 8.4 months. More than three years after splenectomy, at the age of 54, abdominal computed tomography (CT) revealed a continuous thrombus extending from the intrahepatic portal vein to the superior mesenteric vein. In patients with ITP in whom splenectomy fails and treatment with a TPO-RA ± PSL needs to be continued, clinicians should be aware of the possibility of abdominal thrombotic adverse events, such as severe portal vein thrombosis, by following-up on CT imaging, not only in the short term but also in the medium-long term.

## Introduction

Immune thrombocytopenia (ITP) is an autoimmune bleeding disorder affecting approximately 6.1 per 100,000 persons per year in the US population [1]. Since the treatment of ITP has recently changed, the guidelines for the management of ITP have been revised widely internationally [2-4]. In addition to corticosteroid therapy [5] and splenectomy [6], Helicobacter pylori (H. pylori) eradication [7], thrombopoietin-receptor agonists (TPO-RAs) [8,9], and rituximab (RTX) [10] have been recognized as treatment options. However, each treatment has not only advantages but also disadvantages and may have unexpected complications.

Here, we report an instructive case of ITP in which the patient developed severe portal vein thrombosis during oral eltrombopag (a TPO-RA) treatment after splenectomy failure.

## Case presentation

**Figure 1:**
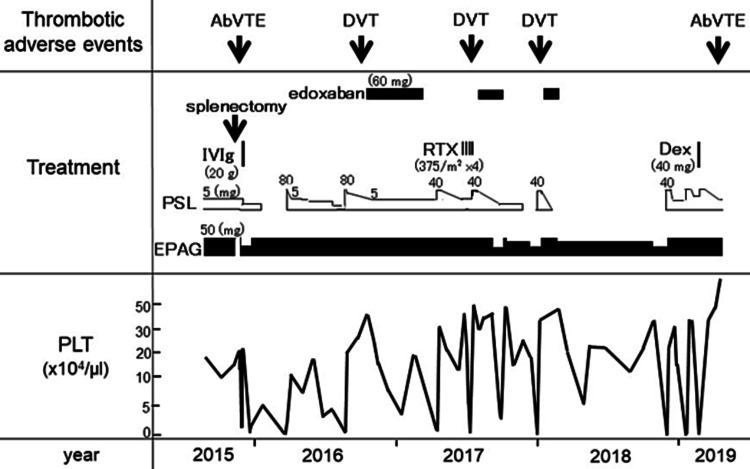
Clinical course after splenectomy Abbreviation: PLT: platelet count; AbVTE: abdominal venous thromboembolism; DVT: deep vein thrombosis; PSL: prednisolone; EPAG: eltrombopag; IVIg: intravenous immunoglobulin; RTX: rituximab; DTX: dexamethasone

A 44-year-old man developed ITP and was followed up at a nearby hospital for two years. Serum H. pylori IgG was negative, and the patient was not receiving any treatment including eradication for H. pylori. At the age of 46 years, the patient visited our department because he developed purpura and nasal bleeding. His platelet count (PLT) decreased to 1,000/μL. He was started on prednisolone (PSL 1 mg/kg: 80 mg). Three months later, the PSL dose was gradually decreased to 15 mg. However, the PLT, which had risen to a maximum value of 101,000/μL, fell to 2,000/μL. Consequently, eltrombopag was started at a dose of 12.5 mg in addition to PSL. Subsequently, eltrombopag was increased to 50 mg, whereas PSL was gradually decreased and discontinued. At the age of 49 years, the patient was suspected to have multiple sclerosis, and methyl-PSL pulse therapy (1 g for three days) was administered at another hospital. Since the PLT dramatically increased to over 700,000/μL, eltrombopag administration was discontinued. However, three weeks later, the PLT decreased to 1,000/μL. Therefore, eltrombopag was resumed and immediately increased to 50 mg. Then, since the patient wanted curative treatment for ITP, we decided to perform a splenectomy. Subsequently, multiple sclerosis was denied. Laparoscopic splenectomy was performed at the age of 51 years (the subsequent clinical course is shown in Figure 1).

Eltrombopag, which the patient had taken for more than four years, except for the abovementioned three weeks, was stopped immediately after splenectomy. Six days after splenectomy, thrombosis occurred in the splenic and portal veins. Low-molecular-weight heparin, warfarin, and urokinase were used for treatment, resulting in the resolution of almost the entire thrombus. Eleven days after splenectomy and the withdrawal of eltrombopag, the PLT decreased to 7,000/μL. High doses of intravenous immunoglobulin (IVIG, 250mg/m^2^ x 3 days) were administered, but the effect was transient (after soaring to over 200,000/μL, PLT will fall again). Although fluctuations in PLT were observed, eltrombopag and PSL were used together for a while. Approximately one year after splenectomy, lower-limb deep vein thrombosis (DVT) was detected. However, no obvious thrombus was observed in the portal vein by abdominal ultrasonography. The patient had no other medical history, such as a history of venous thromboembolism (VTE), surgical history except splenectomy, a family history of venous thromboembolism, or smoking use. In addition, no glucose tolerance or dyslipidemia has been observed to date (August 2021). Thereafter, edoxaban tosylate (60 mg) was administered for five months. Subsequently, although RTX was administered (375 mg/m^2^) weekly for 4 weeks in combination with eltrombopag and PSL for more efficient treatment of ITP, the PLT decreased from 427,000/μL to 6,000/μL. After a while, lower-limb DVT recurred twice, and edoxaban tosylate was administered for a total of 3.4 months. At the age of 54 years, more than three years after splenectomy, the PLT showed a downward trend to 2,000/μL. Therefore, high-dose dexamethasone (40 mg) was administered for four days, while PSL was continuously administered (starting at 40 mg and gradually decreasing). Since then, PLT has increased to over 400,000, but in addition to general fatigue and poor physical condition, the patient showed the following liver function abnormalities: aspartate aminotransferase 132 (normal range: 8-38) U/L, alanine aminotransferase 177 (4-44) U/L, lactate dehydrogenase 459 (120-245) U/L, alkaline phosphatase 434 (105-330) U/L, and γ-glutamyltransferase 152 (<80) U/L.

**Figure 2:**
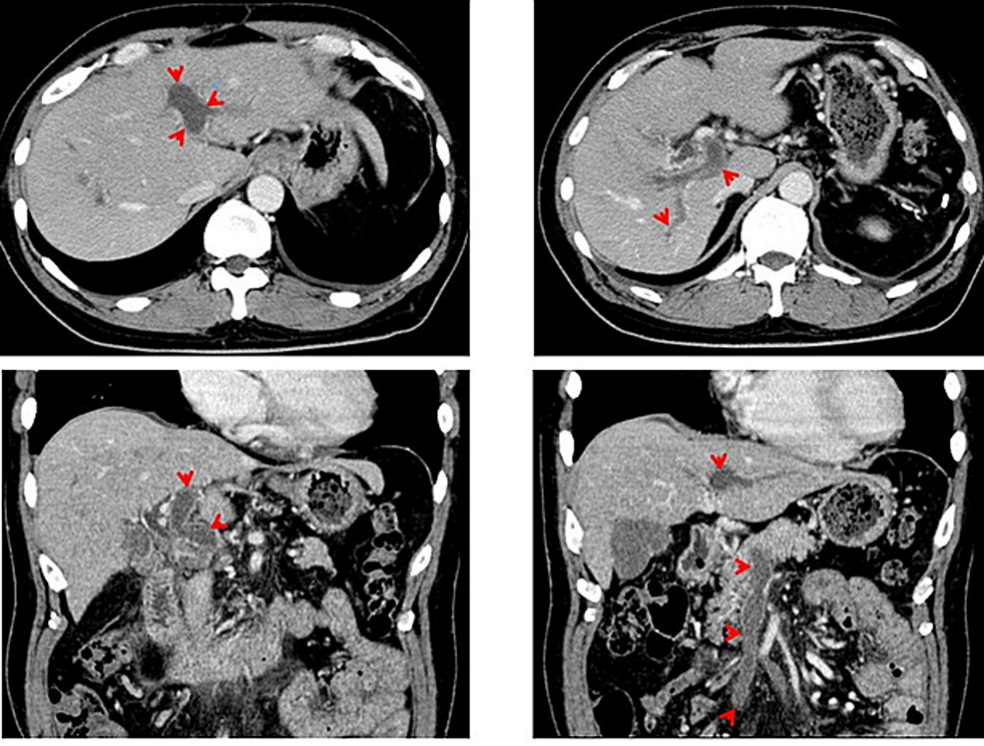
Abdominal computed tomography findings Upper row: The intra-hepatic portal vein was almost occluded by a thrombus (red arrow). Lower row, longitudinal image: The thrombus (red arrow) was also observed in the extra-hepatic portal vein (left) and the superior mesenteric vein (right) continuously (thrombus was also found in the intrahepatic portal vein).

On abdominal computed tomography (CT), a continuous thrombus extending from the intrahepatic portal vein to the superior mesenteric vein was observed (Figure 2). The levels of fibrin degradation products and D-dimer were 11.0 (<5.0 MIC/mL) and 3.99 (<1.0 MIC/mL), respectively. However, there were no abnormalities in the prothrombin and activated partial thromboplastin times or in the levels of fibrinogen, antithrombin III, anti-cardiolipin antibody, lupus anticoagulant, protein C, or protein S. Although not significantly improved by the administration of danaparoid, edoxaban tosylate, and urokinase, the development of abundant collateral vessels supplemented the formation of new portal veins, fortunately, leading to normal activities of daily living without sequelae to date (currently, 57 years old).

## Discussion

This patient was a case of ITP with frequent thrombosis and a marked increase/decrease in PLT. ITP is diagnosed by excluding other diseases because there is no specific test to define it [2]. In a study of a large number of ITP cases treated by experienced hematologists, the two major misdiagnoses were secondary ITP and myelodysplastic syndrome (MDS) [11]. Our patient was followed at a municipal general hospital for the first two years after the onset of ITP. Appropriate examinations, including bone marrow aspiration, were performed at the hospital, and ITP was diagnosed by excluding secondary ITP, MDS, and congenital thrombocytopenia. Furthermore, PLT increased immediately after the administration of IVIG, which made the diagnosis of ITP more accurate [12]. In addition, bone marrow failure syndrome may mainly present with thrombocytopenia [12], and the mean corpuscular volume increases as a distinction from ITP, but our patient has never occurred for 13 years from the time of onset to the present. Since our patient was not taking anticoagulants at the time of the examination for thrombophilia, the data were not a false negative and he does not have thrombophilia.

Regarding the treatment options for ITP, PSL has been a key drug since the 1950s and is still the first-line treatment. Although the long-term administration of PSL has been associated with multiple side effects, PLT decreases with PSL reduction, and only 10%-25% of patients are able to stop PSL treatment [5]. Because several effective treatments are now available for ITP, we started administering eltrombopag three months after the commencement of PSL treatment for this patient.

TPO-RAs bind to the TPO receptor expressed on megakaryocytes, promote megakaryocyte differentiation and maturation, and enhance platelet production [13]; eltrombopag and romiplostim are approved for the treatment of ITP. Both drugs are associated with significant improvements in platelet reactivity and sustained PLT increases [8,9]. In our patient, PLT decreased rapidly over a short period after the discontinuation of eltrombopag following both methyl-PSL pulse therapy and splenectomy. Therefore, the PLT may have been dependent on eltrombopag. Treatment of ITP with TPO-RA may increase the fluctuating of PLT as in our case, which is considered to be a disadvantage [4]. However, we did not consider changing the therapeutic agent in TPO-RA because eltrombopag is not considered to be more severe than romiplostim on the fluctuations of PLT [14]. Approximately half of the cases of arterial or venous thrombosis during the administration of eltrombopag were within one year from the start of treatment and rarely occurred after more than four years [8]. In addition, there are rare reports of portal vein thrombus in hepatitis C virus (HCV)-positive cirrhosis patients using either eltrombopag or romiplostim [15,16]. Although our patient had no history of liver disease, including HCV infection, he developed severe portal vein thrombosis nearly 8 years after starting eltrombopag.

Splenectomy is effective immediately and demonstrates long-term effectiveness of 60%-70% [6]. Therefore, drug treatment is expected to be discontinued after splenectomy, and for this reason, splenectomy has been considered the second-line treatment for ITP [2-4]. The factors that can accurately predict the effectiveness of splenectomy are still unclear, which makes hematologists hesitate to perform splenectomy. With the expansion of the treatment options for ITP, the frequency of splenectomy is decreasing [17]. In our patients, not only did PLT decrease rapidly after splenectomy but unfortunately splenic and portal vein thrombosis also developed. Multiple studies have revealed that splenectomy patients continue to exhibit a slightly increased frequency of VTE, such as lower extremity vein thrombosis (as seen in our patient) and pulmonary embolisms, as compared to nonsplenectomy patients [17,18]. The mechanism may be increased circulation and exposure of damaged red blood cells, cholesterol, and C-reactive protein due to loss of filtering activity of the spleen [19]. On the other hand, Boyle et al. examined postsplenectomy abdominal VTEs (AbVTEs), including portal vein and splenic vein thrombosis, separately from VTE [17]. They investigated 9,976 ITP cases, including 1,762 splenectomy cases, and found that the incidence of AbVTE was 1.6% in splenectomy cases compared to 1% in nonsplenectomy cases less than 90 days after splenectomy. However, no difference in the incidence of AbVTE was observed after more than 90 days [17]. Recently, the usefulness of splenectomy after increasing the PLT with TPO-RA has been reported [20]. In this study, among the 31 patients, only one male patient with a history of HCV hepatitis developed portal vein thrombosis. To avoid postoperative thrombosis, it is essential to establish an appropriate TPO-RA withdrawal period before splenectomy. We did not administer the antithrombotic drugs during the perioperative period of the splenectomy. In our patient, splenectomy was considered to be the direct cause of the thrombotic adverse events in the splenic-portal veins during the postoperative period; however, severe portal vein thrombosis occurred more than three years after the surgery despite the patient taking edoxaban tosylate for a total of 8.4 months, so it was thought due to not only splenectomy but also continued treatment with eltrombopag. A rapid increase in PLT after administration of high-dose Dex and subsequent PSL may also be associated with this complication.

## Conclusions

Our patient developed severe portal vein thrombosis while splenectomy was ineffective and continuing to use eltrombopag. It is a rare phenomenon for patients with ITP. However, clinicians should be aware of abdominal thrombotic adverse events in the medium to long term when splenectomy fails and treatment with a TPO-RA ± PSL needs to be continued, especially if the patient has a history of splenic vein thrombosis after splenectomy. Especially when the PLT increases sharply, it should be recognized that thrombosis may have developed.
